# Optimal final adult height achieved by low-dose recombinant human growth hormone therapy

**DOI:** 10.2478/abm-2024-0011

**Published:** 2024-04-30

**Authors:** Tansit Saengkaew, Suparb Aroonparkmongkol, Suttipong Wacharasindhu

**Affiliations:** Division of Endocrinology, Department of Pediatrics, Faculty of Medicine, Chulalongkorn University, Bangkok 10330, Thailand; Endocrinology Unit, Department of Pediatrics, Faculty of Medicine, Prince of Songkla University, Songkhla 90110, Thailand

**Keywords:** growth hormone, growth hormone deficiency, growth hormone treatment, recombinant human growth hormone, short stature

## Abstract

**Background:**

Thailand has been administering the recombinant human growth hormone (rhGH) treatment for >20 years. Due to limited resources being available, efforts have been directed toward utilizing rhGH at the lowest feasible dose. However, there is currently a lack of evidence in terms of the efficacy and outcomes.

**Objective:**

To evaluate the auxological outcomes of growth hormone (GH) treatment and the GH secretion ability after reaching final adult height (FAH) and discontinuing rhGH.

**Methods:**

Data of 40 patients were retrospectively reviewed. The clinical characteristics, auxological data, and results of biochemical and endocrine investigations before and during rhGH treatment were evaluated. In addition, GH retesting was performed in 24 patients using the insulin tolerance test.

**Results:**

Twenty patients (50%) had complete growth hormone deficiency (GHD), defined as peak stimulated GH level <5 ng/mL, and the remaining patients had partial GHD. Most patients were male (n = 25, 62.5%). The mean age at which rhGH was initiated was 8.9 years. Patients with partial GHD received a higher dose of rhGH than those with complete GHD (30.9 µg/kg/d vs. 26.2 µg/kg/d, *P* = 0.02). Patients with complete and partial GHD reached FAH at height standard deviation scores (SDSs) of −0.65 and −1.47, respectively. The factors associated with obtaining a good clinical response in terms of height gain included peak-stimulated GH level, age of puberty, and age of discontinuing rhGH. After completing the rhGH treatment, 13 of the 24 patients showed normal GH secretion. Patients with multiple pituitary hormone deficiency (MPHD) were likely to have persistent GHD through adulthood (n = 8, 88.9%).

**Conclusion:**

This study has demonstrated that the use of low-dose rhGH could result in healthy populations achieving optimal FAHs. Patients with MPHD might not require retesting as they were likely to have persistent GHD. The results obtained in this research highlight the benefits of the treatment. This treatment can be applied in resource-limited countries.

Recombinant human growth hormone (rhGH) has been developed and widely used to treat children with growth hormone deficiency (GHD) for more than a decade [1,2,3]. Many studies have reported on the optimal clinical outcomes obtained from the treatment such as improving the final adult height (FAH) [4,5,6]; however, the outcomes differ according to the particular population included in each study, ethnicity, growth hormone (GH) cut-off level, and rhGH dosage [4,5,6,7,8,9,10,11]. In addition, rhGH is indicated for treating several conditions besides GHD, including chronic kidney disease, Turner syndrome, *SHOX* gene haploinsufficiency, Noonan syndrome, Prader–Willi syndrome, and idiopathic short stature, as well as non-catch-up small-for-gestational age (SGA) infants. This demonstrates the varying outcomes of rhGH treatment obtained depending on each condition [12].

Several factors have been reported to associate with optimal treatment outcomes, including sex, age, height standard deviation score (SDS) at baseline, mid-parental height (MPH), and peak-stimulated GH levels, combined with other pituitary hormone defects, dosage and duration of rhGH, and height velocity (HV) in the first year of treatment, as have been reported [4, 6, 8, 10, 13, 14]; however, each report shows a difference in the factors involved.

In Thailand, although rhGH treatment has been available for >20 years, there are only a few reports on FAH and other clinical outcomes. In King Chulalongkorn Memorial Hospital (KCMH), one of the largest pediatric endocrine centers in Thailand, rhGH has been used for treating children with GHD since 1995. In 2007, we reported that 20% of short children from our endocrine clinic had GHD, and that half of them had normal GH secretory ability when they reached their FAH [15]. Recently, a number of patients with GHD and treated with rhGH have been observed to reach FAH and discontinue treatment [16]. In resource-limited countries such as Thailand, financial challenges are a primary concern, a fact supported by a previous study in Southern Thailand that found that only 18% of the GHD patients received GH treatment [17]. We tried to use the minimal dosage to optimize the FAH with the lowest cost. Unfortunately, there is no other report available describing the results obtained. Therefore, we aim to evaluate the auxological outcomes of rhGH treatment, including the height-response pattern and FAH, the predictors of good response to rhGH, and the capability of pituitary GH secretion after patients reach FAH.

## Materials and methods

This study was approved by the Ethics Committee, the Faculty of Medicine, Chulalongkorn University (IRB no. 459/63). The consent was waived due to the retrospective study.

In this study, we have retrospectively reviewed the medical records of patients with GHD, who received rhGH treatment from 1995 to 2015 at the Pediatric Endocrine Clinic, KCMH, Bangkok, Thailand. Children with Turner syndrome, Noonan syndrome, or non-catch-up SGA were excluded from this study irrespective of whether they had GHD or not. Data on the baseline clinical characteristics, basic and hormonal laboratory profiles, brain MRI, stimulated GH level, and dosage and duration of rhGH treatment were collected. The primary endpoint was the FAH used to evaluate the effectiveness of rhGH treatment in patients with GHD. FAH was compared to pretreatment height and MPH because it represents the genetic potential of height attainable in each individual.

Clinical data (age, height, weight, HV, pubertal staging, and bone age) and biochemical data (insulin-like growth factor-1 [IGF-1], insulin-like growth factor binding protein-3 [IGFBP-3], free T4, TSH, and peak GH levels]) were retrieved from the medical records. All parameters were collected in a 6-month interval in the first year and annually after treatment until rhGH was discontinued. The dosage of rhGH in this study was calculated based on individual patient requirements during the treatment period. Dose adjustments during rhGH treatment were made by assessing HV followed by IGF-1 levels. Additionally, fasting blood sugar, hemoglobin A1c levels, and lipid profiles were checked to monitor the potential side effects. Puberty was recorded when the testicular volume was >3 mL in boys and when breast buds appeared (i.e., breast Tanner stage II) in girls. All data such as height, weight, and IGF-1 and IGHBP-3 levels were transformed into the SDS according to sex- and age-matched data of Thai children [18].

GHD was diagnosed when children had height or HV <-2 SDS and when the 2 peak stimulated GH levels during provocative tests were <10 ng/mL using the insulin tolerance test, clonidine test, or glucagon test. Abnormal MRI was defined when structural pituitary abnormalities such as hypoplasia, stalk defect, or brain tumor that destroyed the sella turcica area were noted.

Complete and partial GHD were defined as peak GH levels <5 ng/mL and those between 5 ng/mL and 10 ng/mL, respectively, according to previous studies [7, 19]. Other pituitary hormone status and treatments were also recorded.

Additionally, the GH retesting results of patients with GHD, who had discontinued GH treatment after they achieved FAH, were collected to evaluate the GH status after treatment. Transition GHD was defined as a cut-off value of <5 ng/mL according to the adult GHD guideline [20].

### Assays

GH, IGF-1, and IGFBP-3 concentrations were measured by chemiluminescence, using immunoassayIMMULITE® 1000 Immunoassay System (Siemens, Erlangen, Germany). GH intra-assay coefficients of variation (CVs) were 3.5/4.2% at 2.6 ng/mL and 17 ng/mL, respectively; the interassay CVs were 6.5/6.6% at 2.6 ng/mL and 17 ng/mL; and the lower assay sensitivity was 0.15 ng/mL. IGF-1 intra-assay CVs were 6.3/2.4% at 56 ng/mL and 863 ng/mL, respectively; the interassay CVs were 7.6/3.0% at 56 ng/mL and 863 ng/mL; and the lower assay sensitivity was 13.3 ng/mL. IGFBP-3 intra-assay CVs were 4.2/4.8% at 0.91 µg/mL and 8.83 µg/mL, respectively; the interassay CVs were 6.6/5.2% at 0.91 µg/mL and 8.83 µg/mL; and the lower assay sensitivity was 0.1 µg/mL.

### Statistical analysis

Data with normal distribution are expressed as mean and SD. Data without normal distribution are presented as the median with interquartile range (IQR). Comparisons between groups were performed using *t*-test and rank sum test for data with and without normal distribution, respectively. Correlations among variables were analyzed using Spearman’s correlation. *P*-values <0.05 were considered statistically significant. Statistical analysis was performed using the R program (R Foundation for Statistical Computing, Vienna, Austria).

## Results

This study included 40 patients with GHD who were treated with rhGH and who subsequently achieved FAH. Data on the clinical characteristics, investigations, and the details of rhGH treatment at the start and after rhGH was discontinued are shown in **Table 1**. Most patients were male (n = 25/40, 62%); the mean age of the patients was 8.9 years. They exhibited a delayed bone age of 4.5 years at presentation with a lower height SDS (height SDS −2.13) compared to the normal population. The genetic potential height was normal in these patients who presented with an MPH SDS of 0.49. The mean puberty age was 12.2 years in female patients and 13.3 years in male patients. IGF-1 and IGFBP-3 levels were in the normal range, with SDSs of −0.97 and −0.79, respectively. The peak stimulated GH level was 4.63 ng/mL. One fourth of the patients had other pituitary hormone deficiencies: 17.5% had TSH deficiency, 12.5% had ACTH deficiency, 17.5% had diabetes insipidus (DI), and 17.5% had hypogonadotropic hypogonadism. Most patients were diagnosed with idiopathic GHD (n = 33, 82.5%), while the rest of them had pathological causes as observed on abnormal brain MRI, including 6 with craniopharyngioma and 1 with pineal gland tumor.

**Table 1.** Clinical characteristics before and after rhGH treatment categorized according to the GH status as complete vs. partial GHD

	**Overall (n = 40)**	**Complete GHD (n = 20)**	**Partial GHD (n = 20)**	***P***
Sex, n (%)				1.0
Male	25 (62.5)	12 (60.0)	13 (65.0)	
Female	15 (37.5)	8 (40.0)	7 (35.0)	
Baseline				
Age (years)	8.9 (3.0)	9.6 (3.4)	8.2 (2.6)	0.1
Bone age (years)^*^	4.5 [3.0, 7.8]	7.0 [2.8, 9.0]	4.3 [3.3, 4.9]	0.4
Height SDS	−2.13 (0.94)	−2.30 (1.07)	−1.96 (0.78)	0.3
Weight SDS	−1.19 (1.35)	−1.04 (1.50)	−1.34 (1.21)	0.5
HV (cm/year)^†^	3.55 [3.00, 4.80]	3.30 [2.95, 4.08]	4.50 [3.18, 4.93]	0.1
MPH SD	−0.49 (1.0)	−0.16 (0.087)	−0.83 (1.02)	0.03
Peak GH (ng/mL)	4.63 (2.98)	2.56 (1.80)	6.70 (2.44)	<0.001^*^
IGF-1 SDS	−0.97 (1.56)	−1.12 (1.93)	−0.83 (1.11)	0.6
IGFBP-3 SDS	−0.79 (1.44)	−1.08 (1.25)	−0.51 (1.60)	0.3
MPHD, n (%)	10 (25.0)	9 (45.0)	1 (5.0)	0.01^*^
TSH deficiency	7 (17.5)	6 (30.0)	1 (5.0)	0.1
DI	7 (17.5)	6 (30.0)	1 (5.0)	0.1
ACTH deficiency	5 (12.5)	5 (25.0)	0 (0.0)	0.1
Hypogonadism	7 (17.5)	7 (35.0)	0 (0.0)	0.01^*^
MRI finding, n (%)				0.1
Normal	21 (52.5)	9 (45.0)	12 (60.0)	
Abnormal	7 (17.5)	6 (30.0)	1 (5.0)	
Not performed	12 (30.0)	5 (25.0)	7 (35.0)	
Age at puberty (years)^†^	12.8 [11.9, 13.8]	13.1 [12.5, 14.0]	12.0 [11.2, 13.3]	
Male	13.3 (1.7)	13.7 (2.2)	12.9 (0.9)	0.3
Female	12.2 (1.5)	13.2 (0.8)	10.5 (0.8)	<0.001^*^
rhGH treatment				
Duration of treatment (years)	4.9 (3.3)	5.7 (3.2)	4.0 (3.3)	0.1
rhGH dosage (μg/kg/d)^†^	28.4 [21.3, 31.7]	26.6 [19.8, 29.1]	30.9 [28.0, 31.9]	0.02^*^
Discontinue rhGH				
Age (years)^†^	15.3 [14.4, 16.0]	15.3 [14.9, 16.2]	15.3 [14.0, 16.0]	0.5
Bone age (years)^†^	14.4 [14.0, 16.0]	14.4 [14.0, 16.0]	14.5 [14.0, 15.0]	0.9
FAH (cm)				
Male	166.2 (5.9)	168.0 (6.0)	161.4 (1.8)	0.003
Female	152.5 (6.1)	152.2 (7.2)	153.2 (2.8)	0.4
FAH SDS^†^	−0.87 [–1.56, −0.14]	−0.65 [–1.22, 0.70]	−1.47 [–1.66, −0.78]	0.2
Height SDS gain (start-stop of rhGH)	1.43 (1.00)	1.62 (1.09)	0.91 (0.47)	0.1
FAH-MPH SDS	−0.64 (1.38)	−0.55 (1.7)	−0.87 (1.76)	0.6

DI, diabetes insipidus; FAH, final adult height; GH, growth hormone; GHD, growth hormone deficiency; HV, height velocity; IGF-1, insulin-like growth factor-1; IGFBP-3, insulin-like growth factor binding protein-3; IQR, interquartile range; MPH, mid-parental height; MPHD, multiple pituitary hormone deficiency; rhGH, recombinant human growth hormone; SDS, standard deviation score.

† Data are presented as median [IQR], otherwise mean (SD).

* Statistically significant (*P*-value <0.05).

**Figure 1.**
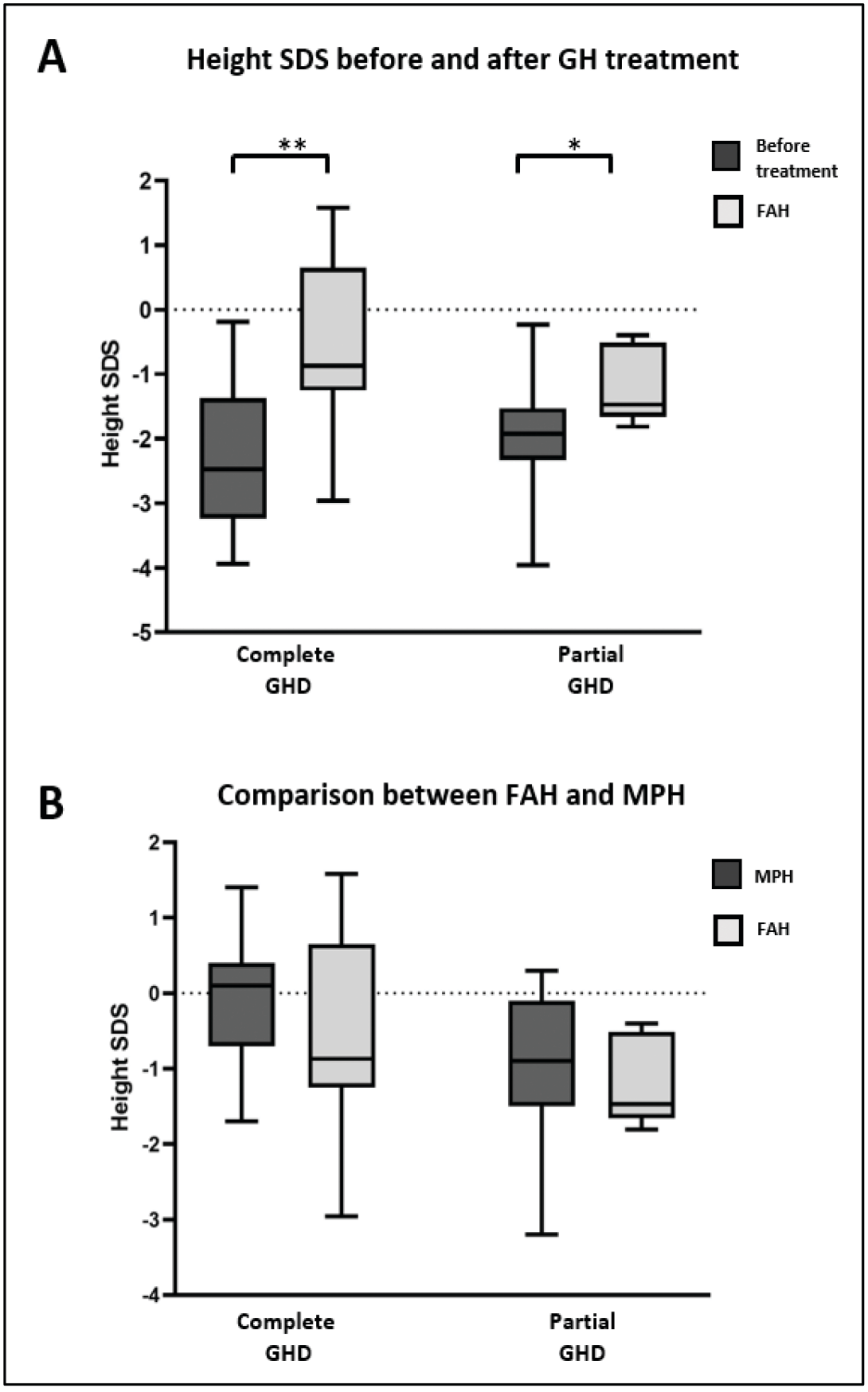
FAH compared to height before rhGH treatment (**A**) and MPH (**B**). The data are presented as median ± IQR. ^*^P < 0.05, ^**^P < 0.005. FAH, final adult height; GH, growth hormone; GHD, growth hormone deficiency; IQR, interquartile range; MPH, mid-parental height; rhGH, recombinant human growth hormone; SDS, standard deviation score.

In this study, patients received rhGH at an average dosage of 28.4 (IQR: 21.3, 31.7) µg/kg/d for 4.9 ± 3.3 years with a mean frequency of 6 d/week. rhGH treatment was discontinued at a mean age of 15.3 years and a bone age of 14.4 years. At the end of the rhGH treatment, patients with GHD reached the FAH at an SDS of −0.87, which was in the normal range of that noted in the Thai population. In this study, we observed that the height SDS (i.e., FAH SDS) at the end of the treatment period is greater than the height SDS observed before the administration of the rhGH treatment. These findings suggest a positive effect of rhGH therapy on FAH outcomes in the treated population. Moreover, the FAH SDS was not different compared with the MPH SDS. These results demonstrated that rhGH treatment in patients with GHD could improve the FAH SDS (*P* < 0.005) (**Figure 1A**), resulting in patients reaching their genetic potential height (**Figure 1B**).

This study also compared the characteristics and outcomes between complete and partial GHD groups (**Table 1**). There were more patients with multiple pituitary hormone deficiency (MPHD) in the complete GHD group (n = 9, 45%) than in the partial GHD group (n = 1, 5%; *P* = 0.01). Additionally, female patients with complete GHD entered puberty later than those with partial GHD (13.2 years vs. 10.5 years, *P* < 0.001). The remaining clinical characteristics and laboratory test results showed no statistically significant differences between the two groups. Interestingly, patients with partial GHD needed a higher rhGH dose than those with complete GHD to achieve normal FAH (26.6 µg/kg/d vs. 30.9 µg/kg/d, *P* = 0.02). Nevertheless, patients in both the groups achieved normal FAH with an SDS of −0.65 and −1.47, respectively, without a statistical difference (*P* = 0.2).

To demonstrate good response to rhGH treatment, we found that peak GH levels (*r* = −0.162, *P* = 0.045), age on entering puberty (*r* = 0.022, *P* = 0.01), and age when rhGH treatment was discontinued (*r* = 0.018, *P* = 0.04) were correlated with the height SDS gain. Patients with lower GH levels at diagnosis tended to have higher height increments after treatment. In contrast, patients with late puberty and delayed age when rhGH treatment was discontinued tended to have higher height SDS gains.

Twenty-four patients were evaluated using ITT to assess GH secretion after completing rhGH treatment at the age of 17.0 ± 2.9 years, which is the transitional period before patients are transferred to adult care. In all, 11 patients (45.8%) showed persistent GHD, and normal GH secretion was subsequently noted in 13 patients (54.2%). Among patients with complete GHD, transient GHD was observed in 46.7% of the patients, and persistent GHD was observed in 53.3% of the patients. Among the patients with partial GHD, there were a higher number of patients with transient GHD than with persistent GHD (66.7% and 33.3%, respectively). Persistent GHD was noted in patients with MPHD compared with those with an isolated GHD (80% and 11.4%, respectively). Only 1 patient with MPHD had transient GHD, while the remaining patients had persistent GHD (**Table 2**).

**Table 2.** Proportion of retesting GH results categorized according to the GH status and hormonal deficiency

	**Persistent GHD (n = 11)**	**Transient GHD (n = 13)**	***P***
GH status, n (%)			0.4
Complete GHD	8 (53.3)	7 (46.7)	
Partial GHD	3 (33.3)	6 (66.7)	
Hormonal defects, n (%)			0.002^*^
Isolated GHD	3 (20.0)	12 (80.0)	
MPHD	8 (88.9)	1 (11.1)	

GH, growth hormone; GHD, growth hormone deficiency; MPHD, multiple pituitary hormone deficiency.

* statistically significant.

## Discussion

Since 1985, rhGH has been widely used for treating children with GHD [2]. Several studies have reported that children with GHD regardless of the etiologies can achieve FAH with differences in the treatment dosage and frequency. In Thailand, rhGH has been used for several decades; however, there are no reports on rhGH treatment outcomes. In this retrospective study, we first reported the primary outcome of rhGH treatment, i.e., FAH and height gain, in patients with GHD who received rhGH treatment. We demonstrated that patients with GHD achieved FAH at −0.87 SDS, which was in the normal range of that noted in the Thai population. The results also showed a significant improvement in height SDS compared with height before treatment. MPH is the best indicator for individual genetic potential height and has been widely used to demonstrate the clinical outcome of rhGH treatment [6, 9, 11, 14]. This study found that more than two-third of the patients could reach the normal genetic potential (MPH ± 2 SD) after being treated with rhGH, which is in the normal height range of the healthy population.

The age of puberty for girls with complete GHD was later than that for girls with partial GHD, and this could have affected gonadotropin deficiency, which is noted in a higher proportion of patients with complete GHD. The age of pubertal induction of these might affect the timing of puberty. Also, the hypogonadotropic hypogonadism patients were initiated on sex hormone-replacement therapy at the age of 13 years for girls and 14 years for boys, corresponding to the diagnosis of delayed puberty. The average dosage of rhGH used in this study was 28.4 µg/kg/d (0.2 mg/kg/week). The partial GHD group needed a higher dose of rhGH than the complete GHD group (30.9 µg/kg/d vs. 26.6 µg/kg/d) (*P* = 0.02). The partial GHD group had lower FAH SDS and height SDS gain after completing treatment than the complete GHD group. Nevertheless, patients with partial GHD were likely to benefit from the treatment by achieving FAH that was almost the genetic potential height (MPH). Moreover, patients with partial GHD had the possibility of developing other conditions such as a constitutional delayed growth and puberty or familial short stature; hence, such patients could not be completely distinguished based on the findings of current GH stimulation tests [21, 22]. The MPH SDS in the partial GHD group is significantly lower than that in the complete GHD group, which might support the notion that they were concurrent with FSS. Unfortunately, in accordance with CDGP, we did not have access to the age of puberty of the parents. However, puberty onset in partial GHD happens a bit later than usual, indirectly suggesting a possible connection with CDGP.

The findings of this study were similar to those of previous studies that showed an improvement following rhGH treatment in terms of increased height SDS. However, the reported FAH varied from −2.2 to −0.23 SDS depending on the etiology of GHD, disease, and study population [5, 6, 8, 9, 14, 23]. A previous study of 401 Swedish patients with GHD revealed that FAH and height gain in patients with severe GHD were higher than those in patients with partial GHD, and patients with partial GHD needed a higher dose of rhGH. These findings are consistent with our results regarding the higher rhGH dose requirement in the partial GHD group. Although patients with complete GHD achieved higher FAH than those with partial GHD, there was no statistical significance in our study. This result will be beneficial for parent counseling regarding treatment outcomes, particularly for patients with partial GHD. The Thai guidelines recommend initiating rhGH at a dose of 35 mcg/kg/d (equivalent to 0.245 mg/kg/week), which exceeds the dosage utilized in our study [24]. Furthermore, when compared to other studies, the majority of them reported an average dosage ranging from 0.2 mg/kg/week to 0.27 mg/kg/week [5, 6, 8, 9, 23]. These pieces of evidence highlight that our study demonstrated the utilization of a lower dosage, specifically focusing on individuals with complete GHD who were administered a dose of 26.6 mcg/kg/d (equivalent to 0.19 mg/kg/week). Although Reiter et al. [14] reported a lower dose of rhGH (0.14 mg/kg/week) compared to our study, it resulted in a lower FAH (−2.2 to −1.75 height SDS) than that reported in our study. Our results are comparable with the research findings in low-income countries [11]. While the findings do not show the maximum FAH, such patients can achieve FAH that is commensurate with their genetic potential.

We also reported an important aspect in the diagnosis of GHD. In the current study, IGF-1 and IGFBP-3 levels were in the normal range. There was an indication that GHD cannot be excluded in the diagnosis of short children with normal IGF-1 and IGFBP levels. This result is supported by a recent meta-analysis that demonstrated 66% and 50% sensitivity and 69% and 79% specificity for IGF-1 and IGFBP-3, respectively, to diagnosis GHD [25]. Therefore, GH stimulation tests remain the gold standard for diagnosing GHD and should be performed in every short child suspected of having GHD regardless of normal IGF-1 and IGFBP-3 levels.

Peak GH levels before starting treatment, delayed age of puberty, and discontinuing rhGH treatment were found to increase height gain in response to rhGH treatment. This finding is consistent with the results of previous studies [9, 11, 13, 26]. When lower GH levels were reported before treatment, a higher height gain was observed in the study between patients with severe and partial GHD [6]. The correlation between FAH and age of puberty was supported by findings of Kurnaz et al. [11] who demonstrated that starting treatment in the pre-pubertal period could increase FAH better than starting treatment in the pubertal period. These results also indicate that a longer treatment duration may be beneficial for final height gain. Moreover, delayed pubertal induction, especially in patients with MPHD who have hypogonadism, might be beneficial for height gain. Other predictive factors such as sex, MPH, dosage of rhGH, and duration of treatment that were shown to affect FAH in other studies were not identified in the current study [9, 11, 14].

During the transitional period, we performed ITT for retesting GH levels as the gold standard method according to the recommendation of the 2019 American Association of Clinical Endocrinologists (AACE) guideline, which used GH <5 ng/mL as the cut-off point to diagnose GHD for transition patients [20]. More than half of the patients (n = 13/24) had normal GH secretion after treatment was discontinued. This finding is similar to that of a previous study that reported that 56% of the patients had normal GH secretion after reaching FAH [27]. In contrast, patients with MPHD are likely to have persistent GHD compared with those with isolated GHD; this finding is similar to that reported by Thomas et al. [21], who demonstrated that 15% of the isolated GHD cases were persistent GHD cases. Our results imply that most patients with isolated GHD had normal GH secretion after they had reached FAH, and thus, there may be no need to reassess the GH status and rhGH can be discontinued as recommended in the updated 2019 AACE guidelines. GH retesting is not necessary for patients with isolated GHD with normal IGF-1 levels [20]. Notably, patients with isolated GHD having an IGF-1 <0 SDS are still recommended to undergo GH retesting, and our study found that 20% of the isolated GHD group had persistent GHD as adults.

Limitations of this study included a small number of patients receiving rhGH treatment due to financial constraints linked to treatment costs. Enhancements in the national health insurance system could lead to increased patient access. Future studies with a larger patient cohort are needed to better assess rhGH efficacy and determine optimal dosage for Thai children. As a retrospective study, this research did not assess the compliance of rhGH use.

In conclusion, although patients are treated with low-dose, short-duration rhGH, they can achieve FAH that is in the normal range of that noted in normal populations and can achieve height SDS that is near the MPH. Notably, individuals with MPHD exhibit persistent GHD throughout their adult life, making GH retesting unnecessary for this patient group.
